# The Impact of the Long-Term Care Insurance on the Medical Expenses and Health Status in China

**DOI:** 10.3389/fpubh.2022.847822

**Published:** 2022-05-06

**Authors:** Yao Tang, Tianran Chen, Yuan Zhao, Farhad Taghizadeh-Hesary

**Affiliations:** ^1^School of Public Administration, Zhejiang University of Finance and Economics, Hangzhou, China; ^2^School of International Business, Southwestern University of Finance and Economics, Chengdu, China; ^3^School of Global Studies, Tokai University, Hiratsuka, Japan; ^4^TOKAI Research Institute for Environment and Sustainability (TRIES), Hiratsuka, Japan

**Keywords:** Long-Term Care Insurance, medical expenses, health status, difference-in-difference method, medical reform

## Abstract

Based on the panel data of China Health and Retirement Longitudinal Study (CHARLS) in 2011, 2015, and 2018, this paper used the difference-in-difference (DID) method to evaluate the implementation effect how the Long-Term Care Insurance (LTCI) policy impacted on the medical expenses and the health status of the middle-aged and elder population. The empirical results show that LTCI has reduced the outpatient and inpatient quantity by 0.1689 and 0.1093 per year, and cut the outpatient and inpatient expenses by 23.9% and 19.8% per year. Moreover, the implementation of LTCI has improved the self-rated health, the activity of daily living (ADL), as well as the mental health. These conclusions verify the implementation value of LTCI system and provide policy implications for the medical reform and the further LTCI implementation in a larger scale.

## Introduction

According to the latest data of the seventh national census released by the National Bureau of Statistics of China in May 2021, the proportion of the population aged 60 and over reached 18.70%, and the proportion of those aged 65 and above reached 13.50%. The proportion changes of the elderly demonstrate that the population aging in China has further deepened. In addition, as the average life span of the population increases, China's elderly population is significantly characterized by aging and longevity, correspondingly the number of the disabled elderly is constantly going up as well. According to the *2018–2019 Survey Report on Long-term Care in China*, the proportion of the severely disabled elderly population reached nearly 5%, with a disability rate of 11.8%. More than 40 million disabled, elderly people are in sore need of medical care and at the same time, the rapidly expanding medical expenses have brought heavy burdens not only to elder individuals, but also their families. Given the severe challenges that may come with the gradual increase of the disabled population, Long-Term Care Insurance (LTCI) has been gradually initiated by the governments. From a worldwide perspective, the long-term care insurance development can be traced back to the Social Insurance Law enacted by the Austrian government in 1956, which legislates the right to health care covering a small part of long-term care liability. Later, the LTCI has been implemented gradually in other countries, until Germany has passed the Long-term Care Act in 1995, when the LTCI system was formally proposed as an independent pillar of the welfare system in Germany.

Learning the experience from other countries, the local government of Qingdao City, Shandong Province of China, took the lead in implementing the LTCI system in the cities and towns under its jurisdiction in 2012, and further expanded the implementation scope of the LTCI system to its rural areas in 2015. The trial LTCI system can be implemented firstly in Qingdao was because the elder population aged above 65 has reached 920 thousand at the end of 2011, accounting 12% and the disable and semi-disable elderly has reached 250 thousand. At that time, the medical security system in Qingdao has already been unable to meet the increasing care need for the aging elderly. Therefore, the nursing services have been separated from the medical services and constituted a part of the trial LTCI system in Qingdao. As to the insured targets, those who have basic medical insurance, no matter the medical insurance for the employed or the general residents in the urban area (extending the scope to the rural area after 2015), were automatically insured with the trial LTCI system. They can make use of the care services of the LTCI system since they have been disabled and in bed for more than 6 months, or completely unable to take care of themselves, or their assessed score of the “Daily Living Ability Assessment” were <60. The LTCI system provides economic compensation for the care services generated during the hospitalization, and provides the integrated care services such as health management, maintenance treatments, basic living care, and functional maintenance (rehabilitation training) etc. through the medical care institutions and nursing centers.

The LTCI treatment and nursing types have been determined according to the disable status. The care services of LTCI system in Qingdao can be mainly categorized as four kinds from the severe disable status to the mild disable status: (1) Special medical care, those who are graded II or above are provided 24-h continuous care services in the designated medical institution; (2) Nursing home care, the institutions integrated treatment and convalesce take the obligations to provide 24-h continuous care services; (3) Home care, door-to-door services provided by the nursing institutions; (4) Patrol care, provided by the nursing institutions (including village clinics) of which the duration and categories are less than the third one. The reimbursement rate of LTCI can be as high as 90% for the insured urban employees (including the retired). For those who have basic resident medical insurance (including rural residents since 2015), the rates are about 70–80% according to the specific care they have accepted. As to the patrol care, the cost such as visiting cost, medical consumable material cost etc. that happens during patrol care are paid by LTCI fund, while the cost such as medicine, examination etc. that happens during hospitalization are still paid by the medical insurance.

After Qingdao being the first city to conduct the trial implementation of the LTCI policy in China, the Ministry of Human Resources and Social Security of the PRC issued the *Guiding Opinions on Piloting the Long-term Care Insurance System* on June 27, 2016, which marks the determination and endeavor of the central government to solve the care problems for the elderly. Since then, another 15 cities across the country has started their trial work of the LTCI system. Several years past, what is the policy effect of the LTCI system? Can it effectively reduce medical expenses of the elderly and their family? Will it have negative effects on the health of the elderly with the reduced medical expenses or have positive effects on their health because the insured elderly patients can access to care services in a better way? In order to answer a series of questions related to the effects of the LTCI policy implementation, a few quantitative studies have been carried out.

By analyzing the long-term care insurance system in the trial cities, scholars concluded that LTCI has lowered the medical expenses and controlled the scale of expenses of the elderly without harming their health (1–3). Using the synthetic control method, Yu et al. (4) found that after implementing the LTCI system, the per capita medical expenses decreased briefly, but later increased. As these studies used the macro data at the municipal level, it was difficult to accurately investigate the long-term impact of LTCI at the micro perspective, for example the medical expenses of elder individuals. Scholars found that the LTCI subsidies for home care significantly reduced their expenses of medical services (5, 6). About every 1 yuan invested in the elderly care system can save about 8.6 yuan of the medical insurance fund (7). These studies contribute the literature on this issues, still there are rare quantitative studies on the effect of trial LTCI policy in China, and the conclusions have been no closer to consensuses. Therefore, based on the 2011, 2015 and 2018 panel data of China Health and Retirement Longitudinal Study (CHARLS), this paper uses the difference-in-difference (DID) method to empirically analyze the effect of the LTCI policy in the experimental cities, exploring whether the original intention of formulating the policy has been achieved and whether it should be implemented nationwide as one of the formal pillars of the social security system of China.

The rest of this paper is structured as follows: As a literature review on LTCI, the Section Literature Review analyzes the impact of LTCI on the medical expenses and the health status of the elderly and proposes the corresponding theoretical hypotheses; Section Data and Recognition Strategies describes the data, samples, model and empirical methods used in this paper; in Section Empirical Results and Interpretation, this paper analyzes the empirical results, conducts the robust test and discusses the mechanism; the Section Conclusions and Policy implications summarizes the whole paper and presents the policy implications for the future implement and medical reform.

## Literature Review

Governments hold the cautious opinions on the LTCI system, worrying the possible increasing care expenses which may be not able to afford. In addition, no consistent conclusions have been reached on the impact of the LTCI policy according to currently available researches, especially its impact on the medical expenses. This paper reviews relevant literature from the following two perspectives.

### The Impact of LTCI on the Medical Expenses of the Elderly

On the one hand, the implementation of LTCI can reduce medical expenses because of its “substitution effect”. Although the development of LTCI are different in different areas, there are many similarities in essence, which basically fall in between the commercial insurance and social insurance. There are neither pure commercial LTCI nor pure state-borne LTCI. Theoretically, with the long-term care services provided, no matter which design of LTCI, the unnecessary utilization of medical services will reduce (8–10). As the LTCI system responds to the much demand for the nursing and care, the burden caused by excessive medical expenses can be eased (11–13). Gade et al. (14) found the medical care providing to the critically ill patients can reduce their use of medical resources, such as the intensive care units, which help to control the medical cost. With the quasi-experimental and randomized controlled trial evidence, researchers have verified that LTCI in U.S can relieve the medical expense burden of elderly patients to some extent (15, 16). Similar research conclusions were found in Japan and South Korea (17, 18). Jae et al. (19) found that patients with LTCI spent less time in hospitals, thus paid significantly reduced medical fees than those patients without LTCI.

On the other hand, some researchers believe that the implementation of LTCI will increase medical expenses because it can release the demand for medical services, namely the “release effect” (20). Jone et al. (21) compared the effect among the different earning groups and concluded the LTCI lead to the higher initial spending on the medical care among middle-income individuals. It is found that the more convenient provision of the medical apparatus and service, the more care services will be utilized for the elderly people (22). Wooldridge and Schore (23) analyzed how channeling impact on the use of hospital, nursing home, and other medical services, and found that the reductions in hospital use among the treatment group were neither large nor statistically significant. Comparing the medical expenses at different ages of the Czech Republic and the Netherlands, the proportion of medical expenses of the elderly who are 85 years old and above is about 20% in Netherlands. In contrast, due to the lack of LTCI in the Czech Republic, the medical expenses of the population over 85 years old did not increase rapidly (4). All the above researches on LTCI indicate that LTCI can release medical needs, thereby increasing the medical expenses of the elderly. However, it would be easy to control the medical expenses simply by closing hospitals, but that would be worthless (24). Only in the way of cutting medical costs without harming the health status of the population, is consistent with the idea of “value-based health care” (25).

### Impact of LTCI on the Health Status of the Elderly

From the macro social perspective, the implementation of LTCI can strengthen the social health welfare because the care services at various levels construct a more complete social security system (26–28). From the micro perspective of elder individuals, research has shown that the degree of care accepted by the elderly is directly proportional to the quality of their longevity and health (29). Based on the experimental results obtained from Spiers et al. (30), cognitive training for the elderly significantly improves their daily activity abilities, which can last for about 5 years. Chen et al. (31) found that the nursing management of the medical institutions and the service attitude of care-givers has been improved with the implementation of LTCI, thus improved the health status of the elderly. Yu and Tseng (32) found LTCI effectively satisfies the nursing demands of the aging society, improves the health status of the elderly, as well as the household caregivers In addition, Fu et al. (33) found the LTCI has a positive effect on labor supply, which had increased after the LTCI system implemented, and had decreased after the LTCI system had reformed. Researchers have realized the critical role of LTCI in improving the health status of elder individuals and their family, members, as well as its positive role in other social field.

## Data and Recognition Strategies

### Data Source

The data used in this paper is extracted from the panel data of CHARLS in 2011, 2015, and 2018, including the micro-data representing families with middle-aged and elderly people over 45 years old. This data is mainly used to analyze the problems of population aging faced by China, which provides a high-quality basic guarantee for the data needed for interdisciplinary research. This paper chooses the CHARLS database for the following reasons: firstly, it is a survey data specifically for the middle-aged and elderly people over 45 years old, which is the target group of the LTCI system, who are potentially taking the care service. Secondly, the LTCI system was first implemented in Qingdao in 2012, and implemented in other 15 cities in 2016. In these 15 cities which implemented the trial LTCI, the data of twelves cities were included in the CHARLS database which are Chengde, Qiqihar, Shanghai, Suzhou, Ningbo, Anqing, Shangrao, Jingmen, Guangzhou, Chongqing, Chengdu, as well as Qingdao. The 2011, 2015, and 2018 CHARLS data contribute to the observation of the situation before and after the implementation of the LTCI policy at different time nodes, which is consistent with the model setting of the DID method. Thirdly, the data covers a series of key variables about medical expenses and the health status of the middle-aged and the elderly, which satisfies the study objects of this article.

### Model and Methods

With the pilot programs of LTCI in different cities as the “natural experiments”, this paper establishes a DID model, with a specific model setting as follows:


(1) $$\begin{array}{l} Y_{ijt}=\alpha +\beta Trial_{ij}\times Treat_{it}+\delta X_{ijt}+\tau_{t}+\omega_{i}+\varepsilon_{ijt} \end{array}$$

As in formula (1), *Y*_*ijt*_ represents the explained variables which this paper emphasized, namely the medical consumption variable (medical expenses and treatment frequency) and health level (self-rated health status and the ability of activities of daily living etc.). *i* represents the individual, *j* represents the cities of trial LTCI, *t* represents time, alternatively the year. *Trial*_*ij*_ × *Treat*_*it*_ represents the interaction term of the city variable whether it had implemented the LTCI and the time variable whether the LTCI had been implemented at the year *t*. The coefficient β is the focus of this study, which is used to observe the policy effect of LTCI. *X*_*ijt*_represents the characteristics of individuals. τ_*t*_ and ω_*i*_ represent the time and individual fixed effect respectively. ε_*ijt*_ represents the random disturbance term.

### Variable Descriptions

Firstly, this paper discusses whether the implementation of LTCI can reduce the medical expenses of the elderly. The dependent variables related to medical expenses, are represented by the outpatient expense, the outpatient frequency, the inpatient expense, and the inpatient frequency in the past year (the logarithms of the variables mentioned above are taken). The main explanatory variable is the impact of LTCI policy, which is the interaction term of dummy variables whether it is the trial cities and the year before and after the implementation of the LTCI system, namely *Trial*_*ij*_ × *Treat*_*it*_. In this paper, the year 2016 is set as the time benchmark. As to the non-trial cities of LTCI, *Trial*_*ij*_ = 0; As to the trial cities of LTCI, Treat_it_ = 1 if the samples are extracted from the CHARLS in 2018, because the LTCI has been implemented; otherwise Treat_it_ = 0 (when the samples are extracted from CHARLS in 2011 and 2015). To the special city Qingdao, which has implemented the LTCI system in 2012, Treat_it_ = 1 if the samples are extracted from the urban area of Qingdao in 2015 and 2018 of the CHARLS database. Otherwise Treat_it_ = 0. (Note: Qingdao has further expanded the implementation scope of the LTCI system to its rural areas in 2015, the rural area is not regarded as the treatment group in 2015, because the interval is about only half a year between the data collection and the LTCI implementation).

Secondly, this paper explores whether the implementation of LTCI will improve the health status of the elderly. The dependent variable, which is the health status of the elderly, is represented by the variables, such as the health self-evaluation score, the ADL score and the mental health score (MHS). The health self-evaluation score ranges from 1 to 5, with 1 point indicating extremely unhealthy and 5 points indicating extremely healthy. Covering six activities of daily life, the ADL score ranges from 0 to 40 points. Similarly, the range of the MHS is also from 0 to 40 points, which is calculated through a set of questions testing the depression symptom of the individuals. For both of the ADL score and MHS, the higher the scores, the worse the conditions. In addition, all variables are standardized before putting in the DID regression analysis.

The control variables are selected based on Anderson's health service utilization model and Grossman's theory of health care demand (34, 35). Grossman's theory of health care demand emphasizes that health investment is necessary to stay healthy, which is impacted by the price of the medical resources. The relatively lower cost of the care services compared with their earnings, provoke individuals to obtain more health resources. In addition, according to Anderson (34), the impacting factors of health service utilization mainly include the personal characteristic factors composed of age, gender, education, earnings, subjective and objective health status etc. Therefore, this paper controls the following variables whose definitions are shown in Table 1.

**Table 1:** Definitions of variables.

**Variable**	**Definition**	**N**	**Mean**	**S.D**.
Age	Age of the elderly	42,591	62.3	13.524
Gender	Dummy variable (male = 1; female = 0)	42,591	0.353	0.496
Marriage	Dummy variable (widowed/divorced = 1; others = 0)	42,591	0.219	0.483
Education	Uneducated = 1; primary school = 2; junior high school = 3; senior high school = 4; college and above = 5	42,591	2.885	2.231
Individual-earnings	Earnings over the past year	42,591	3,211	17,435
Self-rated health	Extremely unhealthy = 1; relatively unhealthy = 2; healthy = 3; relatively healthy = 4; extremely healthy = 5	42,591	3.974	1.213
Disease quantity	The total quantity of chronic diseases suffered by the elderly	42,591	18.95	14.72
Physical pain	Yes = 1; no = 0	42,591	0.402	0.527

*Source: the charls 2011, 2015, and 2018*.

### Descriptive Statistics

As the paper focused on the changes of the medical services utilization and expenses, and the health condition, the mean difference of the related variables before and after the LTCI implementation have been analyzed. The results are shown in Tables 1, 2 respectively.

**Table 2:** The comparison of medical service utilization, 2011, 2015, and 2018.

	**Trial cities**					
	**Before**			**After**		
	**Mean**		**S.D**.	**Mean**		**S.D**.
Outpatient expense (Yuan)	273.5726		898.6157	189.4393^***^		1,126.5433
Outpatient frequency	0.3218		1.0914	0.2278^**^		0.8348
Inpatient expense (Yuan)	1,612.3099		4,893.1747	1,277.6557^***^		4,099.8214
Inpatient frequency	0.1361		0.4733	0.1026^**^		0.3744
N	1,327			653		
	**Non-trial cities**					
	**2011**		**2015**		**2018**	
	**Mean**	**S.D**.	**Mean**	**S.D**.	**Mean**	**S.D**.
Outpatient expense (Yuan)	219.0321^**^	963.7452	247.7403^**^	1,253.9243	265.6823	1,306.4376
Outpatient frequency	0.3974^*^	1.0857	0.4283^**^	1.1695	0.4041	1.0264
Inpatient expense (Yuan)	1,032.8137^**^	6,866.2960	1,384.0247^*^	8,857.2057	1,471.0475	5,798.36769
Inpatient Frequency	0.1385^*^	0.5372	0.1522^*^	0.4795	0.1563	0.4092
N	14,286		15,130		14,127	

*Source: CHARLS 2011, 2015, and 2018*.

*The trial cities were compared before and after the LTCI system implemented; and the trial cities are Qingdao, Chengde, Qiqihar, Shanghai, Suzhou, Ningbo, Anqing, Shangrao, Jingmen, Guangzhou, Chongqing and Chengdu*.

*The non-trial cities are compared in adjacent years and marked on the first ones*.

*, **, and *** *represent significance at the level of 10, 5, and 1%, respectively*.

As demonstrated in the upper part of the Table 2, for the trial cities, the medical services utilization and consumption has changed greatly after the implementation of LTCI policy. The outpatient expenses and frequency, and the inpatient expenses and frequency all decreased with the reform. To those cities which have not implemented the LTCI system, as shown in the lower part of the Table 2, statistical results demonstrate that the medical expenses have increased, no matter the outpatient or the inpatient expenses, and the frequency of the medical services utilization, especially the inpatient frequency also have increased.

The upper part of the Table 3 demonstrated the physical and mental health changes with the implementation of the LTCI system. For the trail cities, the ADL and the MHS of the individuals has improved, even not much. The self-rated health has decreased a little, but not significant. As to those cities which have not implemented the LTCI system, the self-rated health has firstly decreased in 2015, and increase later in 2018. In contrast, the scores of the mental health and the ADL have increased, which means the worse physical and mental circumstance as time goes by.

**Table 3:** The comparison of the health status, 2011, 2015, and 2018.

	**Trial cities**					
	**Before**			**After**		
	**Mean**		**S.D**.	**Mean**		**S.D**.
Self-rated health	3.5313		7.7615	3.5308		6.1343
ADL score	4.3654		11.2351	4.3058^*^		12.7523
Mental health score	17.7856		90.3986	15.7183^***^		75.4348
N.	1,327			653		
	**Non-trial cities**					
	**2011**		**2015**		**2018**	
	**Mean**	**S.D**.	**Mean**	**S.D**.	**Mean**	**S.D**.
Self-rated health	3.7452	7.2358	3.5034^*^	8.243	3.6762	8.7743
ADL score	4.2376	9.4209	4.3458^*^	11.4875	4.3987	12.7541
Mental health score	16.7052^**^	73.0985	17.875^**^	93.7835	18.2423	93.6573
N.	14,286		15,130		14,127	

*Source: CHARLS 2011, 2015, and 2018*.

*The trial cities were compared before and after the LTCI system implemented; and the trial cities are Qingdao, Chengde, Qiqihar, Shanghai, Suzhou, Ningbo, Anqing, Shangrao, Jingmen, Guangzhou, Chongqing and Chengdu*.

*The non-trial cities are compared in adjacent years and marked on the first ones*.

*, **, and *** represent significance at the level of 10, 5, and 1%, respectively.

### Parallel Trend Test

**Figure 1:**
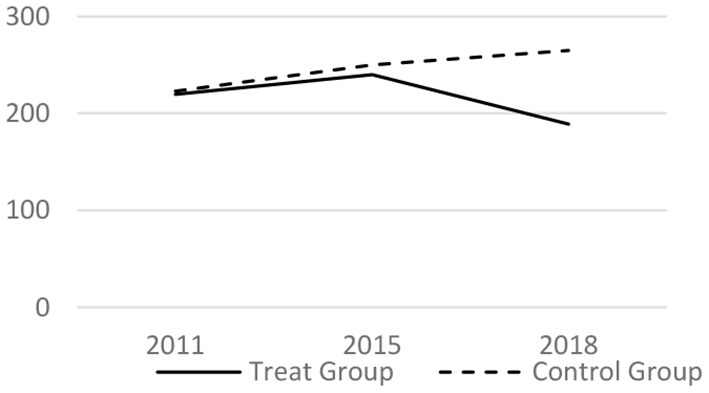
Parallel trend of outpatient expenses (Yuan).

**Figure 2:**
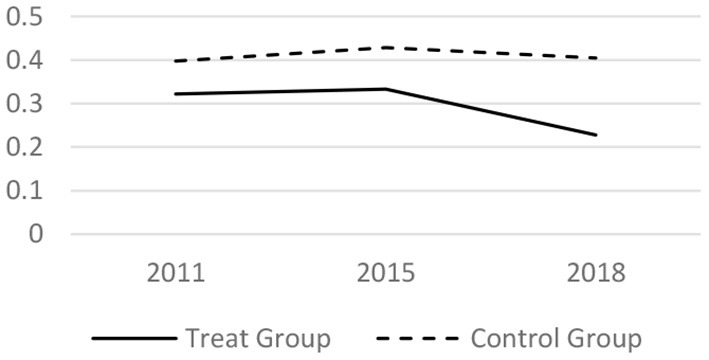
Parallel trend of outexpatient frequency.

**Figure 3:**
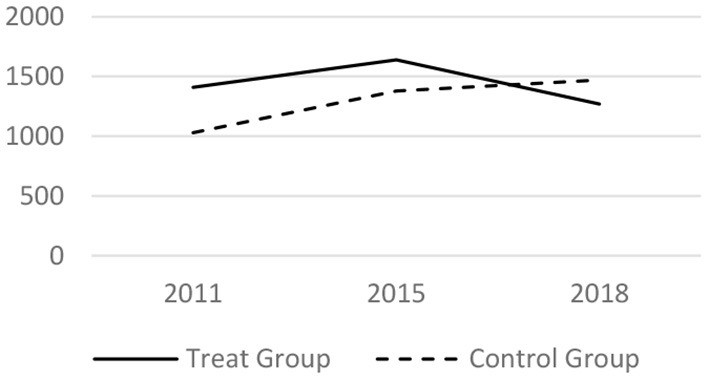
Parallel trend of inexpatient expenses (Yuan).

**Figure 4:**
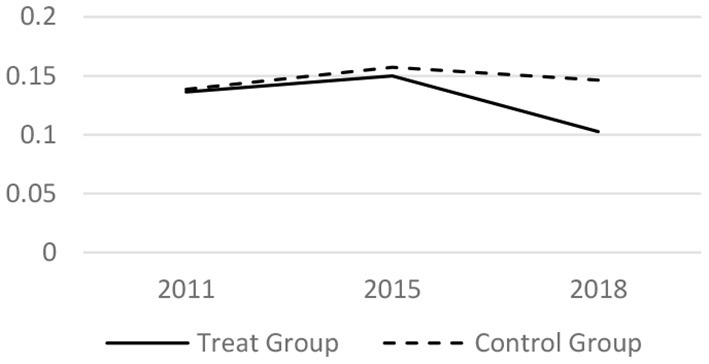
Parallel trend of inexpatient frequency.

**Figure 5:**
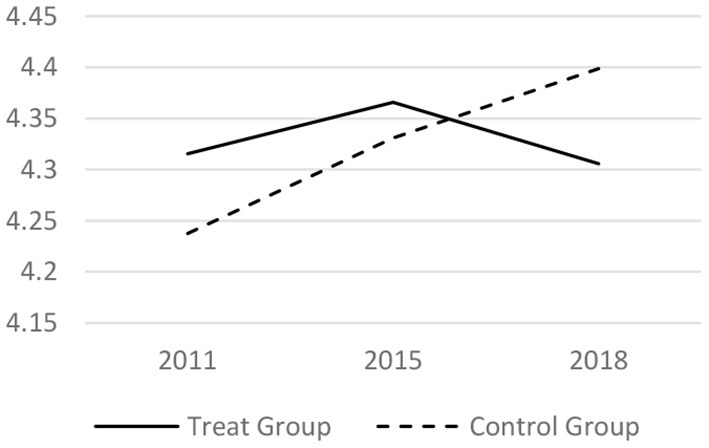
Parallel trend of ADL score.

**Figure 6:**
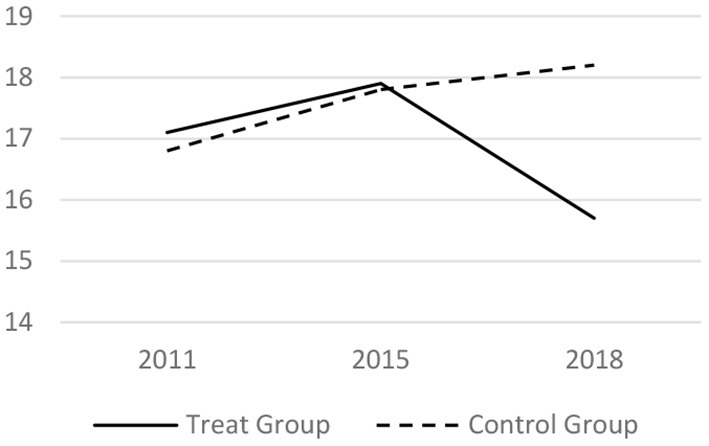
Parallel trend of mental health score.

Before taking the DID regress model, it tested whether the trends before the LTCI implementation is parallel. The core related variables of the medical services utilization and the health conditions in 2011, 2015 and 2018 have been analyzed. The results are shown in Figures 1–6 respectively, which also visually demonstrated how the medical service utilization and health conditions have changed before and after the LTCI implementation.

## Empirical Results and Interpretation

### The Impact of LTCI on Medical Expenses

#### Regression Results of the DID Model

According to the empirical method introduced above, this paper mixes the CHARLS data in three periods (2011, 2015, and 2018) and identifies whether the implementation of the LTCI policy reduces the expenses and frequency of the medical treatment. Table 4 shows the empirical results:

**Table 4:** DID regression results of the expenses and frequency of the medical services.

**Variable**	**(1) lnoutpatient_expense**	**(2) Outpatient_frequency**	**(3) lninpatient expense**	**(4) Inpatient_frequency**
Did	−0.2282^**^	−0.1689^**^	−0.198^*^	−0.1093^**^
	(0.1132)	(0.0786)	(0.1057)	(0.0431)
Age	0.0162^*^	0.0628^**^	0.0693^**^	0.0541^*^
	(0.0097)	(0.0271)	(0.0285)	(0.0312)
Gender	−0.0314	−0.152^**^	−0.0672^*^	−0.253^***^
	(0.0302)	(0.0651)	(0.0399)	(0.0883)
Marriage	−0.0253^*^	−0.0332^*^	−0.0376^*^	−0.0292
	(0.0142)	(0.0181)	(0.0212)	(0.0192)
Education	0.0078^*^	0.0064	0.0077^**^	0.0792
	(0.0045)	(0.0042)	(0.0031)	(0.0727)
Income	0.0083^**^	0.0054^**^	0.0061^*^	0.0045^*^
	(0.0041)	(0.0023)	(0.0035)	(0.0027)
Self–rated health	−0.169^*^	−0.421^***^	−0.147^*^	−0.532^***^
	(0.1012)	(0.1473)	(0.0821)	(0.1609)
Disease_quantity	0.0473^***^	0.0242^**^	0.0524^***^	0.0367^**^
	(0.0182)	(0.0113)	(0.0171)	(0.0154)
_Cons	0.402^*^	−0.0592^**^	0.0221^*^	−0.723^**^
	(0.0653)	(0.0191)	(0.0046)	(0.0924)
N	38728	38728	38728	38728

*Source: charls 2011, 2015, and 2018*.

*The number in the first row of each column is the coefficient and inside the brackets is the standard error clustering to the community level*.

*, **, and *** represent significance at the level of 10, 5, and 1%, respectively.

*The regression has controlled the entity fixed effects and time fixed effects*.

As shown in Columns (1) and (2) of the Table 4, compared with those in non-trial cities of the LTCI system, the implementation of LTCI has reduced the outpatient expenses by 22.82% and decreased the outpatient quantity by 0.1689 per year. It can be seen from Columns (3) and (4) that the inpatient expenses and quantity in the trial city also have reduced (19.8% and 0.1093 respectively). Moreover, among the controlled variables, the age, education and disease quantity are positively correlated with the outpatient and inpatient medical services. Alternatively, those who are elder and with stronger educational backgrounds, higher earnings and more chronic diseases spend significantly more in outpatient medical services, as the coefficients are 0.0162, 0.0078, 0.0083, and 0.0473 respectively. This conclusion can also be applied to the inpatient expenses. In addition, compared with males, the female have significantly higher inpatient expenses and quantity.

#### Robustness Test

##### Robust Test With Reselection of the Dependent Variable

The medical expenses and treatment quantities were used as explanatory variables to study the impact of LTCI system on the medical utilization in trial cities. To verify the robustness of previous conclusions, the related variables such as the reimbursement for the inpatient and outpatient expenses have been taken to replace the explanatory variables mentioned above. As the inpatient and outpatient expenses are not fully covered by the medical insurance, the reimbursement can be used to the check whether the burden of medical consumption has changed before and after the LTCI implementation. The results are demonstrated in Table 5, where the regression coefficients of DID in Column (1) and Column (2) are significantly negative (−0.2461 and −0.2617), indicating that the implementation of LTCI makes the reimbursement of the medical insurance decreased. This is basically consistent with the previous regression results, implying the robustness of the above model. (Note: considering the outliers, sample tail reduction treatment (at top 1% level) have been done and then redo the DID with the same control variables, the basic conclusions did not change).

**Table 5:** DID results with reselection of the dependent variable of reimbursement.

**Variable**	**(1) lnout–patient reimbursement**	**(2) lnin–patient reimbursement**
Did	−0.2461^**^	−0.2617^**^
	(0.0301)	(0.0265)
Age	−0.0819^*^	0.0474^*^
	(0.0423)	(0.0252)
Gender	0.1926^*^	0.1865
	(0.0726)	(0.0843)
Marriage	−0.1206^**^	−0.0224
	(0.0419)	(0.0479)
Education	0.0187^*^	0.0068^*^
	(0.0098)	(0.0037)
Income	−0.0059^*^	−0.0074^***^
	(0.0031)	(0.0028)
Self–rated health	−0.1843^***^	−0.3082^***^
	(0.0538)	(0.1164)
Disease_quantity	−0.0642^***^	0.0442^*^
	(0.0236)	(0.0243)
_Cons	2.1057^***^	1.4133^***^
	(0.0606)	(0.0148)
N	34,948	34,948

*Source: charls 2011, 2015, and 2018*.

*The number in the first row of each column is the coefficient and inside the brackets is the standard error clustering to the community level*.

*, **, and *** represent significance at the level of 10, 5, and 1%, respectively.

*The regression has controlled the entity fixed effects and time fixed effects*.

##### Robust Test With Reselection of the Control Group

The control group analyzed above is composed of all cities that have not implemented LTCI. To further verify the robustness of previous conclusions, we select the second trial cities of LTCI system to form a new control group. Alternatively, Chengde, Guangzhou, Shanghai, Suzhou, Anqing, Shangrao, Jingmen, Chongqing, Ningbo, Qiqihar and Chengdu, were selected from the original control group to constitute a new control group. These 11 cities did not implement LTCI from 2011 to 2015, but they were selected as the trial cities after 2016. These cities may have similar characteristics with Qingdao in terms of the medical service utilization etc., so that they can be chosen to implement the LTCI policy as the second trial tier. Hence, this paper screens the control group to further test the impact of LTCI on the medical expenses and medical treatment quantity, the results of which are shown in Table 6.

**Table 6:** DID results with the second trial tier 11 cities as the control group.

**Variable**	**(1) lnoutpatient_expense**	**(2) Outpatient_frequency**	**(3) lninpatient expense**	**(4) Inpatient_frequency**
Did	−0.1935^***^	−0.0169^***^	−0.2297^***^	−0.0594^***^
	(0.0272)	(0.0058)	(0.0339)	(0.0158)
Age	0.0313^***^	0.0353^***^	0.0468^**^	0.0311
	(0.0119)	(0.0132)	(0.0238)	(0.0218)
Gender	−0.0316^**^	−0.0725^**^	0.0823^*^	−0.142^**^
	(0.0126)	(0.0302)	(0.0497)	(0.0649)
Marriage	−0.0283^*^	−0.0373^*^	−0.0411^*^	−0.295^**^
	(0.0149)	(0.0206)	(0.0231)	(0.1114)
Education	0.0057	0.0027	0.0056	0.0643
	(0.0083)	(0.0021)	(0.0054)	(0.0398)
Income	−0.0023	−0.0015	−0.0057^*^	−0.0036
	(0.0016)	(0.0029)	(0.0032)	(0.0053)
Self–rated health	−0.141^***^	−0.4038^***^	−0.1295^***^	−0.5873^***^
	(0.0105)	(0.0247)	(0.0322)	(0.0357)
Disease_quantity	0.0023^*^	−0.0035	0.0019^*^	−0.0026^**^
	(0.0014)	(0.0026)	(0.0011)	(0.0012)
_Cons	0.0328^**^	−0.0654^***^	0.0416^***^	−0.0877
	(0.0176)	(0.0124)	(0.0031)	(0.1093)
N	2,367	2,367	2,367	2,367

*Source: charls 2011, 2015, and 2018*.

*The number in the first row of each column is the coefficient and inside the brackets is the standard error clustering to the community level*.

*, **, and *** represent significance at the level of 10, 5, and 1%, respectively;

*The regression has controlled the entity fixed effects and time fixed effects*.

As shown in Columns (1)–(4) of Table 6, the regression coefficients of DID are significantly negative (−0.1935, −0.0169, −0.229, and −0.0594 respectively). The regression results are basically consistent with conclusions obtained above which further indicates the robustness of DID model and strengthens the reliability of the conclusion that LTCI can relieve the pressure of the medical services and resources.

### The Impact of LTCI on the Health Status

The health status is represented by the variables of the health self-evaluation score, the ADL score and the MHS. The health self-evaluation score ranges from 1 to 5, the higher the scores, the better the health status. For both of the ADL and MHS, their scores range from 0 to 40 points, which are calculated through a set of related questions. The higher the scores, the worse the conditions. With the DID model, the specific empirical results are shown in Table 7.

**Table 7:** DID regression results of the health status.

**Variable**	**(1) Self–rated health**	**(2) ADL score**	**(3) MHS**
Did	0.1369^**^	−0.0477^***^	−0.0673^***^
	(0.0581)	(0.0249)	(0.0239)
Age	−0.0056	−0.0137^***^	0.1584^**^
	(0.0045)	(0.0041)	(0.0668)
Gender	−0.0302	−0.2156	−0.2436
	(0.0268)	(0.1544)	(0.1736)
Marriage	0.0536^***^	−0.0274^***^	−0.3529^***^
	(0.0143)	(0.0094)	(0.0931)
Education	0.0029	−0.0049^**^	−0.0135^*^
	(0.0023)	(0.0021)	(0.0075)
Income	0.0262	−0.0639^***^	−0.3184^**^
	(0.0492)	(0.0226)	(0.1555)
Cons	0.3173^***^	−0.292^***^	−0.383^***^
	(0.0528)	(0.0936)	(0.1173)
N	38,728	38,728	38,728

*Source: charls 2011, 2015, and 2018*.

*The number in the first row of each column is the coefficient and inside the brackets is the standard error clustering to the community level*.

*, **, and *** *represent significance at the level of 10, 5, and 1%, respectively*.

*The regression has controlled the entity fixed effects and time fixed effects*.

As shown in Column (1) of Table 7, the regression coefficient of DID is positive (0.1369), which indicates that the self-rated health scores in trial cities are higher due to the implementation of LTCI. When it comes to the ADL score and MHS, both coefficients are negative, −0.0477 and −0.0673 respectively. As the higher the scores, the poorer the circumstances, the negative coefficients indicate that the mental health and ability of daily life have improved with implementation of LTCI. Among the control variables, the elder population, the lower health self-rated and ADL scores. Those who are well educated, married and have higher income tend to have significantly better physical and mental health. Alternatively, their scores of the ADL and mental health are lower.

#### Robust Test

##### Robust Test With Reselection of the Dependent Variable

To verify the robustness of the conclusions achieved above, the related variables such as “whether the body is in pain” as the proxy variable to replace the explanatory variables mentioned above. The results are demonstrated in Table 8, where the regression coefficient of DID is significantly negative (−0.1242), indicating that the implementation of LTCI help relieve the physical pain in trial cities. This is basically consistent with the previous regression results, implying the robustness of the above model.

**Table 8:** DID results with reselection of the dependent variable of pain.

**Variable**	**(1) Pain**
Did	−0.1242^***^
	(0.0248)
Age	0.0217^*^
	(0.0112)
Gender	−0.171^**^
	(0.0763)
Marriage	−0.0391^***^
	(0.0132)
Education	0.0011
	(0.0035)
Income	0.0156
	(0.0501)
Cons	−1.825^***^
	(0.1772)
N	32,985

*Source: CHARLS 2011, 2015 and 2018*.

*The number in the first row of each column is the coefficient and inside the brackets is the standard error clustering to the community level*.

*, **, and *** represent significance at the level of 10, 5, and 1%, respectively.

*The regression has controlled the entity fixed effects and time fixed effects*.

##### Robust Test With Reselection of the Control Group

To verify the robustness of previous conclusions that LTCI system has improved the health status of the population, we select the second trial cities of LTCI to constitute a new control group. Alternatively, the sample of 11 trial cities in 2011 and 2015 is the control group. The method is similar with the test on the medical service utilization and the results are shown in Table 9.

**Table 9:** DID results with the second trial tier 11 cities as the control group.

**Variable**	**(1) Self–rated health**	**(2) ADL score**	**(3) MHS**
Did	0.3473^***^	−0.2012^***^	−0.2361^***^
	(0.1275)	(0.0719)	(0.0634)
Age	−0.0064^**^	0.0338	0.0153
	(0.0031)	(0.0797)	(0.0832)
Gender	−0.0289	−0.0297	−0.0328
	(0.0225)	(0.0165)	(0.0732)
Marriage	0.0643^***^	−0.0217^*^	−0.0593^*^
	(0.0132)	(0.0129)	(0.0355)
Education	0.0081	−0.0049	−0.0084
	(0.0055)	(0.0042)	(0.0057)
Income	0.0226	−0.0542^**^	−0.0628
	(0.0692)	(0.0183)	(0.0846)
Cons	4.613^***^	−0.3362^***^	−0.5817^***^
	(0.4896)	(0.0927)	(0.1137)
N	2,335	2,335	2,335

*Source:charls 2011, 2015, and 2018*.

*The number in the first row of each column is the coefficient and inside the brackets is the standard error clustering to the community level*.

*, **, and *** represent significance at the level of 10, 5, and 1%, respectively.

*The regression has controlled the entity fixed effects and time fixed effects*.

The basic conclusions keep the same as seen from the first line of the column (1), (2) and (3) in Table 9. The coefficient of DID is 0.473, which is positive with the dependent variable of self-rated health, indicating that the health status has improved with the implementation of LTCI system in the trial cities. As to the ADL score and MHS, both coefficients are negative (-0.0231 and−0.0226). The negative coefficients indicate that the mental health and ability of daily life have heightened with implementation of LTCI. All these conclusions are consistent with the previous DID regression results.

## Conclusions and Policy Implications

China has entered the aging society since 2000 and has gradually become the country which has the largest aging population in the world. The health care demand for the elderly is estimated to a high level which will break out massive, crises by 2030 as the number of disabled elderly people keep increasing. Against such a backdrop, the central government of China has tentatively implemented the Long-Term Care Insurance System in 15 cities trying to solve the elderly care problems. This paper evaluate the implementation effect of the LTCI policy, basing on the panel data of CHARLS in 2011, 2015, and 2018. Statistics analysis demonstrated that the policy intentions on the medical utilization and the health status have been basically achieved. The empirical results from the DID analysis showed that: on the one hand, as to the medical expenses and utilization, the outpatient and inpatient frequency and expenses has decreased with the implementation of LTCI; on the other hand, the self-rated health, the ADL, especially the MHS has significantly improved in the trial cities, and these conclusions are valid under a series of robust tests.

The implementation of LTCI system separated the nursing services from the medical services, making the medical funds play a bigger role and better health outcomes. These findings provide a useful reference for further deepening medical reform. Firstly, the medical service utilization, such as the hospitalization, is one of the determinant factors on the health and longevity of the population. China should continue to focus on improving the construction of the LTCI system, and at the same time, to reduce the unnecessary excessive medical treatments. In addition, it is necessary to integrate the graded diagnosis and treatment with the LTCI system, making the medical and nursing system mutually matched, as well as in line with China's national condition.

Secondly, the traditional Chinese medical philosophy thinks highly of that the best way to cure a disease is to cure it before it become a disease. Since keeping health is the fundamental goal of the medical system reform, and the regularly patrol care and health examinations can help maintain their physical and mental health, the medical examination services and health education activities needed to be expanded, especially to those patients with chronic diseases. Last but not least, in order to strengthen the health management of the middle-aged and elder population, professional care service practitioners are indispensable. Faced with a huge shortfall in care service practitioners, training mechanism of the care service team to improve their professional service skills are also important. In sum, to construct the LTCI system is just the first step to deal with the huge need of elderly care services with the increasing expanding aging population.

## Data Availability Statement

The CHARLS databases are public and open to researchers who has applied for them. The data can be applied at: http://charls.pku.edu.cn/, further inquiries can be directed to the corresponding authors.

## Author Contributions

YT and TC: conceptualization, methodology, and writing—original draft. YT and YZ: formal analysis. FT-H: investigation and validation. YZ and TC: software. TC and FT-H: writing–review and editing. All authors have read and agreed to the published version of the manuscript.

## Funding

The research was funded by the National Social Science Foundation of China, titled: The Cost Estimation and the Policy Research on the Informal Care for the Disabled Elderly in Rural China (No: 19CSH071) and Zhejiang Provincial Natural Science Foundation Project, titled: Study on the Demand and the Supply of Elderly Care Services and its Multiple Effects in Zhejiang Province (No: LQ20G030021), and the Grant in Aid for Excellent Young Researcher of the Ministry of Education of Japan.

## Conflict of Interest

The authors declare that the research was conducted in the absence of any commercial or financial relationships that could be construed as a potential conflict of interest.

## Publisher's Note

All claims expressed in this article are solely those of the authors and do not necessarily represent those of their affiliated organizations, or those of the publisher, the editors and the reviewers. Any product that may be evaluated in this article, or claim that may be made by its manufacturer, is not guaranteed or endorsed by the publisher.

## References

[1] Zhang C Giles J Zhao Y. Evaluation of the effect of new rural social endowment insurance policy: income, poverty, consumption, subjective welfare and labor supply. Econ Q. (2015) 14:203–30.

[2] Feng J Yu Y Lou P. Medical demand and the growth of medical cost in china: based on the difference of medical cost between urban and rural elderly. Soc Sci China. (2015) 85–103.

[3] Gong X Zhou W. Government subsidies, insurance payments and end-of-life care costs: an analysis based on CLHLS data from 2002 to 2014. South Econ J. (2018) 2018:68–85.

[4] Yu X Liu H Yan W. The impact of long-term care insurance on medical costs. Insur Res. (2019) 2019:114–27.

[5] Li L Yang Y. the impact of population aging on health care costs: a case study of Beijing. Soc Secur Res. (2017) 27–39.

[6] Yao H. Comparison reflection of china's long-term care insurance system under the background of aging crisis. Soc Secur Res. (2020)2020:48–56.

[7] Wang Z Feng J. The Effect of long-term care insurance on medical cost substitution and the comparison of different compensation models. Econ Q. (2021) 21:557–76.

[8] Bei L Yan MH Lim GJ Feng G. Substitution effect of long-term care to hospital inpatient care?. China Econ Rev. (2020) 2209–26.

[9] Costa-Font J Jiménez-Martín S Cristina V. Does Long-term Care Subsidization Reduce Hospital Admissions and Utilization? J Health Econ. (2018) 58:43–66. 10.1016/j.jhealeco.2018.01.002 29408154

[10] Dong J Smieliauskas F Konetzka RT. Effects of Long-Term Care Insurance on Financial Well-Being. (2015). Available online at: https://ssrn.com/abstract=2719776 (accessed December 21, 2015). 10.1057/s41288-018-00113-7 PMC11974404 40196839

[11] Johri M Beland F Bergman H. International experiments in integrated care for the elderly: a synthesis of the evidence. Int J Geriatr Psychiatry. (2003) 18:108–21. 10.1002/gps.819 12642892

[12] Kattenberg M Bakx P. Substitute services: a barrier to controlling long-term care expenditures. Eur J Ageing. (2020) 2020:85–97. 10.1007/s10433-020-00570-x 33746684 PMC7925780

[13] Forte PE. The American long term care insurance program: a solution to reduce cost and provide stability. Generations. (2017) 40:40–53. Available online at: https://chinesesites.library.ingentaconnect.com/content/asag/gen/2017/00000040/00000004/art00008

[14] Gade G Venohr I Conner D McGrady K Beane J Richardson R H. Impact of an inpatient palliative care team: a randomized control trial. J Palliat Med. (2018) 11:180–90. 10.1089/jpm.2007.0055 18333732

[15] Penrod JD Deb P Dellenbaugh C Burgess JF Zhu CWL Christiansen CL. Hospital-based palliative care consultation: effects on hospital cost. J Palliat Med. (2010) 13:210–23. 10.1089/jpm.2010.0038 20642361

[16] McCarthy IM Robinson C Huq S Philastre M Robert FL. Cost savings from palliative care teams and guidance for a financially viable palliative care program. Health Serv Res. (2015) 50:217–36. 10.1111/1475-6773.12203 25040226 PMC4319879

[17] Zhao C Sun J. The reform of long-term care insurance system in japan and its enlightenment. Populat J. (2018) 40:79–89.

[18] Hashimoto HH. Micro data analysis of medical and long-term care utilization among the elderly in Japan. Int J Environ Res Public Health. (2010) 7:3022–37. 10.3390/ijerph7083022 20948944 PMC2954565

[19] Jae CW Park E Lee SGP. Does long-term care insurance reduce the burden of medical costs? a retrospective elderly cohort study. Geriatr Gerontol Int. (2018) 18:207–19. 10.1111/ggi.13536 30311345

[20] Arrizumi H. Effect of public long-term care insurance on consumption, medical care demand, and welfare. J Health Econ. (2008) 27:1423–35. 10.1016/j.jhealeco.2008.07.008 18757107

[21] Jone CR Done N Gerard F. Anderson. Considering Long-term Care Insurance for Middle-income Countries: Comparing South Korea with Japan and Germany. Health Policy. (2015) 119:1319–29. 10.1016/j.healthpol.2015.06.001 26117093

[22] Motel-Klingebiel A Tesch-Roemer C Von Kondratowitz HJ. Welfare states do not crowd out the family: evidence for mixed responsibility from comparative analyses. Aging and Society. (2015) 25:863–82. 10.1017/S0144686X05003971

[23] Wooldridge J Schore J. The effect of channeling on the use of nursing homes, hospitals, and other medical services. Health Serv Res. (2018) 23:78–91. Available online at: https://pubmed.ncbi.nlm.nih.gov/3130323/3130323 PMC1065492

[24] Einav L Finkelstein A Mahoney N. Provider incentives and healthcare costs: evidence from long-term care hospitals. Econometrica. (2018) 86:2161–219. 10.3982/ECTA15022 31130738 PMC6529222

[25] Porter E. What is value in health care? N Engl J Med. (2010) 363:2477–81. 10.1056/NEJMp1011024 21142528

[26] Leena F Mari A Jutta P Jani R Pekka R Marja J. Long-term care is increasingly concentrated in the last years of life: a change from 2000 to 2011. Eur J Public Health. (2017) 49–61. 10.1093/eurpub/ckw260 28339763

[27] Li F Otani J. Financing elderly people's long-term care needs: evidence from China. Int J Health Plan Manag. (2018) 33:479–88. 10.1002/hpm.2488 29327366 PMC6032836

[28] Zhu M. The analysis of demand for long term care and its insurance system constructing in China. Chinese Journal of Health Policy. (2009) 35–44. Available online at: http://en.cnki.com.cn/Article_en/CJFDTOTAL-ZGWZ200907009.htm

[29] Klimaviciute J Pestieau P. Long term care social insurance: how to avoid big losses? Int Tax Publ Fin. (2018) 99–139. 10.1007/s10797-017-9445-4

[30] Spiers G Matthew FE Moffatt S Barker RO. Impact of social care supply on healthcare utilisation by older adults: a systematic review and meta-analysis. Age Ageing. (2018) 2018:107–21. 10.1093/ageing/afy14710.1093/ageing/afy147 30247573 PMC6322507

[31] Chen L Zhang X Xu X. Health insurance and long-term care services for the disabled elderly in China: based on CHARLS data. Risk Healthc Policy. (2020) 2020:155–62. 10.2147/RMHP.S233949 32161509 PMC7051854

[32] Tsu-Wei Y Tseng L. Examining the role of commercial long-term care insurance in long-term care services. Int J Bank Market. (2019) 37:565–78. 10.1108/IJBM-01-2018-0007

[33] Fu RN. Spillover effect of japanese long-term care insurance as an employment promotion policy for family caregivers. J Health Econ. (2017) 56:103–12. 10.1016/j.jhealeco.2017.09.011 29040896

[34] Anderson R. A Behavioral Model of Families' Use of Health Services. Research Series No. 25. Chicago, IL: Center for Health Administration Studies; University of Chicago (1968).

[35] Grosman M. The Demand for Health: A Theoretical and Empirical Investigation. New York, NY: National Bureau of Economic Research. (1972).

